# *NTRK* Fusions in Central Nervous System Tumors: A Rare, but Worthy Target

**DOI:** 10.3390/ijms21030753

**Published:** 2020-01-23

**Authors:** Alessandro Gambella, Rebecca Senetta, Giammarco Collemi, Stefano Gabriele Vallero, Matteo Monticelli, Fabio Cofano, Pietro Zeppa, Diego Garbossa, Alessia Pellerino, Roberta Rudà, Riccardo Soffietti, Franca Fagioli, Mauro Papotti, Paola Cassoni, Luca Bertero

**Affiliations:** 1 Pathology Unit, Department of Medical Sciences, University of Turin, 10126 Turin, Italy; alessandro.gambella@gmail.com (A.G.); giammarco.collemi@gmail.com (G.C.);; 2 Pathology Unit, Department of Oncology, University of Turin, 10126 Turin, Italy; rebesenetta@gmail.com (R.S.); mauro.papotti@unito.it (M.P.); 3 Pediatric Onco-Hematology Unit, Department of Pediatric and Public Health Sciences, University of Turin, 10126 Turin, Italy; svallero@cittadellasalute.to.it (S.G.V.); franca.fagioli@unito.it (F.F.); 4 Neurosurgery Unit, Department of Neurosciences, University of Turin, 10126 Turin, Italy; mmonticelli89@gmail.com (M.M.); fabio.cofano@gmail.com (F.C.); pietro_zeppa@yahoo.it (P.Z.); diego.garbossa@unito.it (D.G.); 5 Department of Neuro-Oncology, University and City of Health and Science Hospital, 10126 Turin, Italy; alessia.pellerino@unito.it (A.P.); rudarob@hotmail.com (R.R.); riccardo.soffietti@unito.it (R.S.)

**Keywords:** central nervous system, glioma, pediatric tumors, molecular pathology, NTRK, gene fusion, targeted therapies, precision medicine

## Abstract

The neurotrophic tropomyosin receptor kinase (*NTRK*) genes (*NTRK1*, *NTRK2*, and *NTRK3*) code for three transmembrane high-affinity tyrosine-kinase receptors for nerve growth factors (TRK-A, TRK-B, and TRK-C) which are mainly involved in nervous system development. Loss of function alterations in these genes can lead to nervous system development problems; conversely, activating alterations harbor oncogenic potential, promoting cell proliferation/survival and tumorigenesis. Chromosomal rearrangements are the most clinically relevant alterations of pathological *NTRK* activation, leading to constitutionally active chimeric receptors. *NTRK* fusions have been detected with extremely variable frequencies in many pediatric and adult cancer types, including central nervous system (CNS) tumors. These alterations can be detected by different laboratory assays (e.g., immunohistochemistry, FISH, sequencing), but each of these approaches has specific advantages and limitations which must be taken into account for an appropriate use in diagnostics or research. Moreover, therapeutic targeting of this molecular marker recently showed extreme efficacy. Considering the overall lack of effective treatments for brain neoplasms, it is expected that detection of *NTRK* fusions will soon become a mainstay in the diagnostic assessment of CNS tumors, and thus in-depth knowledge regarding this topic is warranted.

## 1. Introduction

Traditionally, tumor diagnosis and prognostic evaluation, as well as therapeutic management, were addressed by histological examination alone, which was based on tumor morphology and complementary immunohistochemical profiling. Nowadays this approach is no longer adequate for complete tumor characterization since molecular profiling has become necessary for optimal patient management [1,2,3,4,5]. As a result, diagnostic algorithms are undergoing substantial changes for many tumor types: this molecular revolution has been fully undertaken by the latest 2016 World Health Organization (WHO) classification of central nervous system (CNS) neoplasms, as molecular markers (e.g., *IDH1*/*IDH2* (Isocitrate dehydrogenase 1/2), 1p/19q codeletion, *ATRX* (transcriptional regulator ATRX), *TP53* (tumor protein p53) etc.) have become mandatory for a conclusive diagnosis of many specific tumor entities [6,7,8,9]. Moreover, in the following few years since its publication, the diagnostic/prognostic/predictive importance of many additional molecular traits have been demonstrated and they are now being quickly translated into the routine clinical practice [10,11,12].

Despite the rarity, neurotrophic tropomyosin receptor kinase (*NTRK*) alterations recently gained attention because of the impressive therapeutic results achieved through their specific targeting. Since *NTRK* fusions have been found at significant frequencies in CNS tumors, which typically lack effective therapies, their detection is expected to soon become a mainstay in the diagnostic assessment of these tumors, and specific expertise in this topic will become mandatory.

In this Review, we will discuss the biology and physiological role of TRK receptors as well as their role in pathological conditions, focusing on the recently collected knowledge in brain tumors.

## 2. Biology of TRK Signaling

### 2.1. Characteristics of NTRK Genes and of TRK Signaling 

Tyrosine receptor kinases are a group of cell-membrane high-affinity receptors sharing similar structures and intracellular signaling pathways, but with different mechanisms of activation and regulation. These receptors have specific growth factors as ligands and are involved in several fundamental functions for cell survival and activation, such as growth, differentiation, and apoptosis [13,14,15,16]. The oncogenic role of their alterations is well documented, as well as their possible exploitation as therapeutic targets [17,18,19,20,21,22,23,24,25]. 

*NTRK* are part of this group, consisting in a family of genes (*NTRK-1*, *NTRK-2*, and *NTRK-3*) located on chromosomes 1 (1q22), 9 (9q22), and 15 (15q25) and encoding for the TRK-A, TRK-B, and TRK-C proteins, respectively [26]. They were first identified and described as oncogenes in colorectal cancer by Pulciani et al. in 1982 [27], and then recognized as high-affinity neurotrophin receptors in 1989 [28]. They present the canonical structure of tyrosine kinase receptors, consisting of an intracellular domain with tyrosine-dependent kinase activity linked through the transmembrane structure to an extracellular domain made of two immunoglobulin-like high-affinity receptors and three leucine-rich motifs, the latter being specific of the *NTRK* family [13,14]. Specific neurotrophins, a subset of growth factors, are the main ligands of TRK proteins. TRK-A is probably the most studied and well-characterized receptor of the *NTRK* family and is preferentially bound by the nerve growth factor (NGF) [29]. Neurotrophin-3 (NT-3) binds TRK-C, while TRK-B has a lower binding specificity since both brain-derived growth factor (BDNF) and neurotrophin-4 (NT-4) can be ligands of this receptor [30,31,32,33,34]. Furthermore, also p75NTR, a membrane receptor, member of the tumor necrosis factor (TNF) receptor family, binds all the spectrum of neurotrophins described above and plays a crucial role in balancing cell survival versus death during CNS development [35]. Indeed, these last ligand-receptor relationships should be considered of low affinity [36,37]. p75NTR can also be considered a sort of “sparring partner” of TRK receptors, since their coexpression can enhance the activity of TRKs by improving the affinity between each TRK receptor and the corresponding ligands [38,39]. 

**Figure 1:**
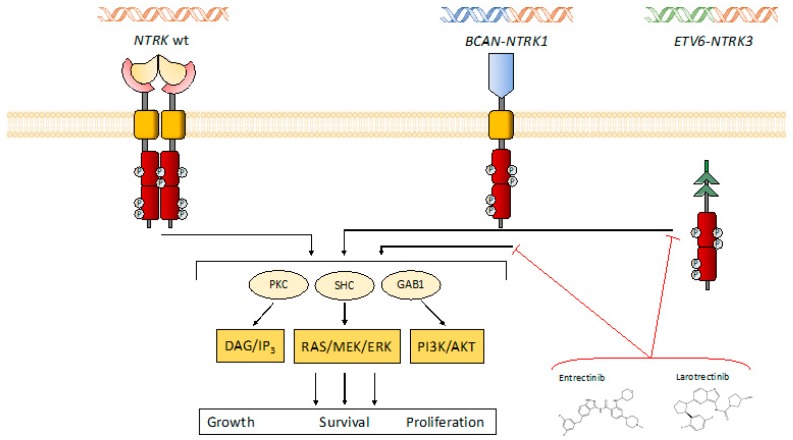
Physiological and rearranged *NTRK* genes/TRK receptors and intracellular signaling. The PLC-*γ*, MAPK, and PI3-K intracellular pathways (here represented by the DAG/IP3, RAS/MEK/ERK, and PI3-K/AKT components, respectively) are activated either from the wild-type form of *NTRK*, and the chimeric fusion receptors (e.g., *BCAN*-*NTRK1* and *ETV6*-*NTRK3*). However, the latter happens in a ligand-free constitutively activated fashion, leading to oncogenic activation. The *NTRK* inhibitors (TKI, here represented by entrectinib and larotrectinib) achieve their antitumor activity by interacting with the intracellular domain of the chimeric receptors, inhibiting the recruitment of the signaling pathway.

TRK receptors activation by their ligands leads to homodimerization of the intracellular domain, followed by phosphorylation of several tyrosine residues and consequent activation of the downstream signaling cascades (Figure 1). So far, TRK-A tyrosine residues have been thoroughly defined (Y496, Y676, Y680, Y681, and Y791) and TRK-B and TRK-C show a similar intracellular domain and activity. The intracellular domain, once phosphorylated, engages at least three different signaling cascades: the Ras-mitogen-activated protein kinase (MAPK), the phospholipase C-*γ* (PLC-γ), and the phosphatidylinositol 3-kinase (PI3-K) pathways. The final result of these interactions causes the activation of the neural cells, enabling their development and maintenance [40,41]. 

Another important signal transduction mechanism of TRK signaling is represented by the endocytic pathway. After binding with their respective partners, TRK receptors can be internalized within signaling endosomes which then can be transported back to the cell body where they can exert their function [42,43]. This mechanism, although it has been demonstrated for multiple receptor types, is especially relevant for neurons, since the cell soma can be significantly distant from the axon extremity. In particular, it has been shown that both signaling at the distal axon extremity and the retrograde trafficking of TRK-A bound with NGF are both necessary for neuronal survival and development.

Isoforms have been described for all three TRK, resulting from splicing variants of the *NTRK* genes and lacking specific subsets of exons [41]. Despite the consequent structural modifications, these isoforms keep the ability to transduce the signal once the ligand is bound [44,45,46]. However, each specific isoform presents peculiar characteristics both in terms of expression (e.g., expression in different tissues or with different timings) and activity [47,48,49,50]. 

### 2.2. The Physiological Role of NTRK Signaling and Its Role in Non-Neoplastic Diseases

**Figure 2:**
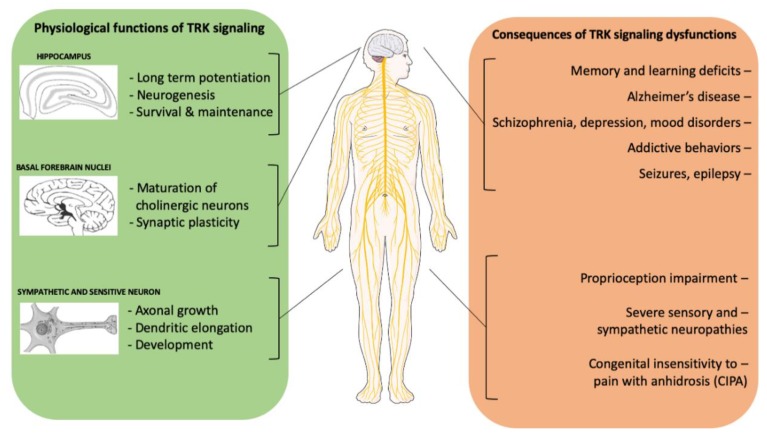
Neurophysiological functions of TRK signaling and possible consequences of its alterations.

The role of *NTRK* in the nervous system has been widely investigated (Figure 2): overall, TRK-B is probably the most represented receptor (mainly located in cortex, cerebellum, striatum, and hippocampus), while TRK-A and TRK-C show more restricted expression profiles, the former being limited to mature forebrain cholinergic neurons, and the latter mainly observed during neuronal development [51,52]. TRKs activation promotes and regulates the growth and elongation of dendrites and axons regardless of the site of origin and of the specific function [52,53]. 

In the peripheral nervous system (PNS), neutrophins binding and *NTRK* signaling is required for the survival of sensory and sympathetic ganglia [52,53,54,55,56]. Loss of *NTRK* expression in the development phase of mice and zebrafish has several consequences on their sensory systems, such as gustatory deficits and hearing and vision impairment [57,58]. Within CNS, *NTRK* gene expression is fundamental for neuron migration to the cortical layer (in particular for cerebellar granule neurons), and for their growth and maturation. Moreover, the hippocampal long-term maturation is strictly associated with *NTRK* expression by resident neurons [55,59,60,61,62].

Because of the significant role played in the physiology of nervous system development and maintenance, the level of expression of *NTRK* genes has been widely studied in pathological non-tumoral CNS conditions [63]. A significant downregulation of these receptors has been observed in the frontal cortex and in cholinergic basal nuclei of patients with Alzheimer’s disease. Moreover, a truncated isoform of TRK-B receptor resulted more expressed than the complete isoform in the cerebral cortex and the hippocampus of these patients. TRK-B truncated isoform lacks the intracellular tyrosine-dependent kinase domain, leading to a non-functional receptor [64].

Also, altered TRK signaling has been suggested in schizophrenia. Although the limited understanding of the pathogenetic mechanisms behind this disorder and the presumptive involvement of multiple genes, the 15q25 region has been identified as a possible culprit. This locus includes the *NTRK3* gene: dysfunction of the corresponding TRK-C receptor could impair neural connections, plasticity, and development of the hippocampus and of the prefrontal cortex in these patients, together with a reduction of the overall levels of neurotrophins [65,66,67].

*NTRK* alterations have been proposed in many other neurological and psychiatric conditions, ranging from epilepsy (where increased levels of BDNF and of TRK-B resulted correlated with seizure induction and severity) to depression and addictive behaviors [67,68]. Additional data regarding the role of *NTRK* alterations in non-neoplastic diseases are now available, but this topic falls outside the scope of the present review.

## 3. *NTRK* in Tumor Development 

### 3.1. The Oncogenic Role of NTRK: Fusions Versus Other Alterations

The oncogenic activation of *NTRK* can occur in several ways, including structural chromosomal rearrangements leading to gene fusions, splice variants, mutations, copy number alterations and increased expression. Considering their clinical relevance, these alterations can be clustered into two main groups: *NTRK* gene fusions leading to constitutively activated receptors versus the other mechanisms. Importantly, these other types of alterations are overall more frequent than NTRK fusions, but they cannot be effectively targeted with the currently available drugs (with the important exception of the *NTRK* mutations developed as a mechanism of resistance to therapeutic inhibition of *NTRK* fusions) and thus are presently considered non-druggable [69,70]. 

Regarding fusions, more than fifty *NTRK* fusion partners have been reported so far, confirming the extremely promiscuous nature of this rearrangement. Nevertheless, the same type of gene structural rearrangement is preserved: the 3′ region of the *NTRK* gene is fused with the 5′ region of a partner gene. The resulting chimeric protein keeps the *NTRK* tyrosine kinase domain with the ability to activate the usual intracellular pathway, but it becomes ligand-independent thanks to the partner gene component. The fusion mechanism described above for *NTRK* oncogenic activation is comparable to those occurring in other oncogenes with a kinase-domain component, such as *ALK* and *ROS1* [71,72,73,74]. Indeed, gene fusions of receptor tyrosine kinases is a common oncogenic mechanism shared by multiple tumor types and leading to oncogene addiction, although the specifically involved genes can vary between the different neoplasms. For instance, if we consider non-small cell lung cancer, fusions involving *ALK*, *ROS1*, *RET*, *BRAF*, *EGFR,* and *NTRK* have been reported [75].

Overall, *NTRK* fusions seem to be rarely present (<1%) in unselected large series of tumors; conversely, it can be practically considered a pathognomonic marker of specific rare neoplasms including breast secretory carcinomas, mammary analogue secretory carcinoma of the salivary glands, infantile fibrosarcomas and congenital/infantile mesoblastic nephroma, narrowing a 100% prevalence [76,77,78,79,80]. Of interest, tumors harboring *NTRK* fusions often (>50%) present other genomic co-alterations in genes related to the *NTRK* intracellular pathways, such as the MAPK and the PI3K signaling cascades, *TP53*-associated genes, cell-cycle regulatory proteins and other tyrosine kinases, although strong mitogenic/driver alterations are usually mutually exclusive [70,81].

The true oncogenic potential of non-fusion *NTRK* alterations, such as mutations, gene amplifications and alternative splicing has yet to be confirmed [49,50,82,83,84,85,86]. Moreover, as it will be furtherly discussed later, these alternative types of alterations can play a crucial role in tumor resistance against *NTRK*-fusions inhibitors and therefore are being increasingly investigated [70,87].

### 3.2. NTRK Alterations in Non-CNS Tumors

Considered that *NTRK* was discovered as a potential oncogene in colorectal cancer (CRC) [27,88], and that tumors in which *NTRK* fusions can be considered pathognomonic belong to non-CNS cell-lineages as well, the oncogenic potential of this signaling pathway is not restricted to tissues with *NTRK* physiological expression [76,77,78,79,80]. 

Non-CNS *NTRK*-altered tumors include neoplasms with high incidence, but low frequency of *NTRK* fusions and rare tumors with extremely low incidence, but high frequency of this molecular hallmark. In this latter group, assessment of *NTRK* fusions can be used also as a diagnostic marker. 

Among the first group, which represents the sharp majority of cases, *NTRK* fusions occur in no more than 4% of CRCs and the detected fusions so far are the *TPM3*-*NTRK1* [89,90,91], the *LMNA*-*NTRK1* [92], and the *ETV6*-*NTRK3* [93]. CRC harboring *NTRK*, *ALK,* and *ROS1* could be distinctively identified as tumors with high frequency of metastasis, poor prognosis, and specific mutational profile, characterized by high microsatellite instability (MSI) and *RAS* and *BRAF* wild-type status [70,94]. These observations can help aim *NTRK* assessment in this setting. The second main tumor type within this group is lung adenocarcinoma, in which NTRK rearrangements occur in about 3% of lesions. The main observed fusions are *CD74*-*NTRK1*, *MPRIP*-*NTRK1*, and *TRIM24*-*NTRK2* [72,95]. Another carcinoma harboring *NTRK* as a potential oncogene is papillary thyroid carcinoma (PTC), with at least two different fusion products, *TPM3*-*NTRK1* [96,97] and *ETV6*-*NTRK3* [98].

The second group includes three entities, namely secretory carcinoma (either arising from breast or from salivary glands), congenital/infantile fibrosarcoma and congenital mesoblastic nephroma [76,99,100,101]. All these tumors present an *NTRK* fusion in more than 95% of cases, usually the *ETV6*-*NTRK3*. This fusion derives from a chromosomal translocation, *t*(12;15) (p13;q25), which combines exon 4, 5, or 6 of *ETV6* and the kinase domain of *NTRK3* [102]. 

Considering that the potential therapeutic efficacy has been demonstrated across all tumor types, *NTRK* fusions have been evaluated in several other neoplastic entities, ranging from Spitz tumor and melanoma to sarcomas (especially in the pediatric population), pancreatic cancer and cholangiocarcinoma, and neuroendocrine tumors [103,104,105,106,107,108,109]. Variable frequencies of *NTRK* rearrangements have been observed, but they usually are <10%. 

### 3.3. NTRK Fusions in Pediatric CNS Tumors

In Europe and North America, the outcome landscape of pediatric tumors has recently changed: CNS neoplasms overtook hematological neoplasms as the leading cause of death within this population mainly because of the limited efficacy of the available treatments [110,111]. For this reason, pediatric CNS tumors represent an unmet need in oncology, requiring novel approaches for management and treatment. 

Pediatric diffuse low and high-grade gliomas are undergoing significant changes in terms of diagnostic assessment, due to the increasing importance of molecular markers for classification and stratification [10,112,113]. These tumors also harbor peculiar molecular profiles which vary significantly from adult tumors, even in cases with similar histological features. Whenever a definitive diagnosis is achieved, the clinical behavior is still very heterogeneous and tumor recurrences are frequent even after multi-modal integrated treatments [112,114,115,116,117]. In particular, high-grade pediatric gliomas are associated with very limited outcomes and the possible treatments, which include radiotherapy, can lead to severe toxicities in children. A druggable target, like *NTRK* or other fusions, can thus actually have a major impact in this setting, improving disease control and allowing to delay other treatments with less favorable risk/benefit profiles [118].

*NTRK* alterations have been widely described in pediatric gliomas, both in low-grade and high-grade lesions (Table 1). Pilocytic astrocytoma (PA) is the most common pediatric glioma and usually shows a good outcome, especially after complete surgical resection; however, since recurrences do occur, it has been thoroughly investigated to look for new potential therapeutic targets including druggable fusions. MAPK pathway is commonly altered in PA and *BRAF* is the most frequently involved gene (e.g., *KIAA1549*: *BRAF* fusion, *BRAF* V600E mutation), while *KRAS*, and *NF1* mutations can be observed in rare cases. Recently, *NTRK* fusions have also been observed in rare supratentorial PA with involvement of the *NTRK2* gene [112,114,119].

Pediatric high-grade gliomas (pHGGs) are rare lesions with a dismal survival rate: the two-year survival rate for patients with supratentorial pHGGs range from 10 to 30 percent, and it is even lower (<10%) for diffuse intrinsic pontine gliomas (DIPGs). In this unsatisfactory scenario, molecular profiling of pHGGs seems imperative to improve the outcome of these patients by exploitation of specific therapeutic targets. As expected by their heterogeneity in terms of morphological features and clinical behavior, their molecular analysis showed a wide and challenging landscape, with several aberrant pathways and multiple mechanisms of tumor initiation/promotion being concurrently present. Although these findings are usually associated with intrinsic resistance to targeting of single alterations, a particular subset of non-brainstem high-grade gliomas has been identified in younger children (less than three years old) with high frequencies (up to 40%) of *NTRK* fusions (*TPM3-NTRK1* and *ETV6-NTRK3*) without significant additional alterations, opening up new treatment scenarios for these selected cases [117,120,121,122,123]. Nevertheless, the overall *NTRK*-fusion rate of almost 4% observed in unselected cohorts of pediatric gliomas suggests its routine diagnostic assessment [70]. 

So far, *NTRK* fusions have not been detected in ependymoma, another frequent pediatric tumor. Although chromosomal rearrangements are key drivers of this neoplasm (e.g., RELA-fusion), NTRK signaling is not likely to be specifically involved based on the available data [120].

*NTRK* rearrangements have been investigated and discovered with a notable frequency in mixed glioneuronal tumors, a rare group of pediatric epileptogenic CNS neoplasms. Once again, although rare, *NTRK* (particularly *NTRK1*) fusions have been identified in both low-grade and high-grade glioneuronal tumors, ranging from ganglioglioma to diffuse leptomeningeal glioneuronal tumors [124,125,126,127]. 

Medulloblastoma, one of the most common and highly aggressive CNS non-glial pediatric tumors, showed no *NTRK* fusions. However, increased expression of non-mutated receptors, TRK-C in particular, has been found to be associated with a better clinical outcome and prognosis, suggesting the potential exploitation of *NTRK* signaling as a prognostic rather than predictive marker [128,129,130,131].

### 3.4. NTRK Fusions in Adult CNS Tumors

CNS tumors represent a challenging context also among adults with discouraging outcomes. Comprehensive molecular analyses of large cohorts of these tumors have been conducted, focusing on high grade gliomas (HGG) and glioblastoma (GBM), the latter being the most common glioma in adults with an extremely severe prognosis. IDH-wildtype GBM (the so-called primary glioblastoma) shows a broad spectrum of potentially targetable alterations, including a significant rate of fusions: chimeric fusion genes are often present, and involvement of all of the three *NTRK* genes has been demonstrated (Table 1), although with significant differences among the series [132,133]. Up to date, *NTRK2* appears to be the most frequently involved gene (up to 11% of GBM), while *NTRK1* fusions are definitely rarer (about 1%) and *NTRK3* fusions seem to be extremely rare (one single case reported) [133,134,135,136,137]. Among low grade gliomas (LGG), a *NTRK1* fusion was reported in an adult pilocytic astrocytoma [133]. 

Expression and methylation of wild-type *NTRK* genes has been investigated in different types of gliomas as well, revealing that LGGs present higher expression of TRK receptors compared to HGGs. Although these findings need to be further confirmed, lower expression levels in tumoral cells seem to be associated with increased malignant potential and poorer prognosis [138,139]. Accordingly, higher expression of *NTRK* receptors in neuroblastomas was found to be associated with a better outcome. This finding is possibly due to immunoregulatory mechanisms, thus widening the potential range of modulatory effects associated with this signaling pathway [140].

## 4. *NTRK* as a Novel Therapeutic Target

### 4.1. NTRK-Fusions Targeting: A Novel, Effective, Histology-Independent Anti-Neoplastic Treatment

Drug development in oncology has significantly changed since the discovery of targetable molecular alterations. Since these alterations are shared among completely independent tumor sites and types, basket trials were initiated, testing cohorts of patients with common molecular targets, despite the different tumor entity [141]. However, in some cases (e.g., mutated *BRAF*-inhibitors) response to treatments was still histology or tissue-dependent and thus drug approval was limited to specific indications. More recently, pembrolizumab, an anti-PD1 immunomodulatory drug, received tissue-agnostic approval considered the efficacy in a wide range of advanced tumor types sharing mismatch repair deficiency or high microsatellite instability. Similarly, *NTRK*-inhibitors are receiving tissue-agnostic (FDA) or histology-independent (EMA) approval based on high efficacy in pediatric and adult tumors harboring *NTRK* fusion regardless of the tumor site or specific fusion partner. So far, two first-generation molecules (entrectinib and larotrectinib) received FDA therapeutic approval for the treatment of *NTRK* fusion-positive tumors and the latter recently gained EMA approval as well [41].

Entrectinib (RXDX-101) was the first drug developed against *NTRK* fusions, targeting also *ALK* and *ROS1* fusion proteins and harboring a good delivery rate through the blood-brain barrier [142]. In phase-I and II trials (ALKA-372-001, STARTRK-1, STARTRK-2, and STARTRK-NG), it showed significant results in pediatric and adult solid tumors, with efficacy in both primary and secondary CNS tumors [69,143]. In a recent series of pediatric high-grade gliomas reported at ASCO 2019, all 4 patients achieved a radiological response, including a complete response (2019 ASCO Annual Meeting, Abstract #: 10009).

Larotrectinib (LOXO-101) is highly specific for *NTRK* fusions only, and its efficacy has been tested in several trials (registered on ClinicalTrials.gov: NCT02637687, NCT02122913, NCT02637687, and NCT02576431) [144,145], with a well-documented efficacy against CNS tumors [145], as recently confirmed [146]. 

These results are important for two main reasons: (i) the overall rate of clinical and radiological responses is high (even close to 80%); (ii) response is usually durable, with patients achieving disease control for many months or even years.

Ongoing clinical trials with entrectinib and larotrectinib are now focused on elucidating their activity profile (e.g., to assess possible correlations with the specific fusion partners) and safety data (Table 2). Moreover, development and clinical testing of second-generation *NTRK* inhibitors is already ongoing (e.g., repotrectinib-TPX-0005 and LOXO-195-BAY2731954) [147,148], in order to compare their efficacy with first-generation drugs and, more importantly, to tackle tumor resistance to them.

### 4.2. Resistance Mechanisms to First-Generation NTRK Inhibitors

Acquired resistance during long-term treatment with targeted therapies is a major concern, as experienced with *EGFR*, *ALK*, and *ROS1* inhibitors [149,150,151,152,153,154]. *NTRK* inhibitors make no exception to this statement, and disease progression has been now observed within the ongoing clinical trials. 

Notably, excluding sporadic cases whose failure was related to non-appropriate patient recruitment [155], at least two broad mechanisms of resistance have been detected. The first one is related to off-target alterations, which reactivate one of the cellular pathways associated with *NTRK* fusions, usually the MAPK. As a matter of fact, MAPK signaling cascade may get activated by several signal transducers not related to *NTRK* at all. Examples of this resistance mechanism are the acquisition of the *BRAF*^V600E^ or *KRAS*^G12D^ mutations or *MET* amplification. Of note, in these cases, prompt treatment with drugs targeting the new resistance-related alterations enabled new tumor responses [156]. 

The second tumor escape strategy (the so-called on-target resistance) is related to point mutations (i.e., solvent front, gatekeeper and xDFG mutations) of the *NTRK* fusion proteins, blocking drug binding. In this regard, next-generation *NTRK* inhibitors (e.g., repotrectnib—TPX-0005, LOXO-195-BAY2731954) have been developed, showing promising efficacy in targeting these mutated fusion proteins [87,155,157].

These data open several questions that will be answered by the upcoming trials: can resistance to first-generation inhibitors be avoided by modulating the treatment over time? Should patients directly receive second-generation inhibitors? How should patients be monitored during treatment to promptly detect resistance?

## 5. Testing for *NTRK* Fusions. Where Is Waldo? 

Based on the previous considerations, *NTRK* fusions must now be considered an important molecular marker in CNS tumors, which can enable significant improvement of patients’ outcome by specific targeting. So, how can we efficiently test for these alterations taken into consideration their rarity?

*NTRK* oncogenic activation is a process that, starting from the chromosomal rearrangement, requires translation of the fusion gene and expression of the chimeric TRK protein. In light of these consequential steps, different laboratory assays can be used to find out whether a tumor is harboring a *NTRK* fusion (Table 3). Firstly, to investigate the DNA status, fluorescence *in-situ* hybridization (FISH) and DNA-based next-generation sequencing (NGS) can be used, while reverse transcription-polymerase chain reaction (RT-PCR), real time-PCR and RNA-based NGS analyses can evaluate the transcribed RNA. Finally, immunohistochemical staining (IHC) can directly assess the protein product.

### 5.1. Immunohistochemistry

IHC is a common, well-known and validated assay, with limited costs, and quick turnaround time (TAT), allowing histological correlations and also capable of intrinsically confirming the protein expression. The main limitation is that available antibodies are for the wild-type epitopes of TRK receptors, thus not specific for fusions and obviously they do not provide any information regarding the fusion partner. Different TRK antibodies are available, staining either single receptors (so far, antibodies for TRK-A and TRK-B are available as well as a cocktail of anti-TRK-A and anti-TRK-B) or all TRK proteins (pan-TRK antibody, clone EPR17341), which is also available for in vitro diagnostics (Ventana Medical Systems Inc., Tucson, AZ, USA). Of note, different staining patterns can be expected based on the involved genes: for example, *NTRK3* fusions more often lead to a nuclear staining and a higher rate of false negatives (up to 45%) [77,78,79,80,106,158,159]. IHC can thus be used as an effective screening tool for most tumor types, but unfortunately specificity in CNS neoplasms seems to be low due to the physiological expression of *NTRK* in neural tissues. For instance, Solomon et al., reported an unsatisfactory specificity value of 20.8% in gliomas [160], thus screening by IHC should be avoided in this setting or used with extreme caution and confirmation by other techniques is warranted. A significant rate of false positive IHC results has also been observed in cases with smooth muscle or neuroendocrine differentiation and in small round cell tumors. Of note, in false positive samples, staining was limited to cytoplasm and/or cell membrane without nuclear staining.

### 5.2. Fluorescence In-Situ Hybridization

FISH-based assays are well-established to investigate chromosomal alterations, such as translocations, deletions, or amplifications, thus they could also be applied for evaluating *NTRK* fusions. Considered their promiscuous nature, break apart probes must be used which do not provide information on the fusion partner. Although, as it is true for IHC, FISH requires minimal formalin-fixed paraffin-embedded material and enables a low TAT, a specific expertise for a correct interpretation is required. Moreover, since investigation of all three *NTRK* genes requires three independent assays, a FISH-based approach cannot be envisaged for screening [161]. On the other hand, FISH has been suggested as a confirmatory assay with high sensitivity and specificity, although evaluations of larger series of *NTRK*-fusion tumors are warranted to assess potential limitations. In particular, if the fusion breakpoint is non-canonical, a false negative result can be observed.

### 5.3. DNA and RNA Molecular Testing

The second group of assays that can be used to look for *NTRK* fusions is based on extraction and analysis of nucleic acids. These techniques vary significantly in terms of complexity, costs, TAT (which is usually longer than IHC or FISH), required material, information provided, and thus optimal indications. DNA or RNA can be successfully tested by different assays, but with some important caveats: (i) RNA is more prone to be damaged, especially in FFPE material, thus special attention must be payed to pre-analytics; (ii) RNA-based assays usually require simpler analyses as intronic regions have been already removed; (iii) DNA-based assays can detect rearrangements which are not even transcribed and thus lack any relevance, while, conversely, they can miss fusions involving large intronic regions [162].

Considering the specific techniques, RT-PCR can be used for orthogonal validation of a specific fusion, but fusion partners must be already known, and specific primers must be designed, thus, it cannot be used for screening despite the low costs. Real-time PCR-based assays are now progressively becoming available, allowing assessment of a wide range of combinations of specific rearrangements/partners through an overall inexpensive analysis. The main limitations of this approach are that it usually does not provide information regarding the specific fusion partners and, since it evaluates a pre-determined set of rearrangements, rare or novel fusions will not be detected. 

Regarding NGS, DNA assays are becoming routinely used in diagnostics as they can assess a wide range of clinically relevant alterations (mutations, copy number variations, tumor mutation burden), but reporting time and costs are significant, and specific facilities and expertise are required. Chromosomal rearrangements can also be detected, but sensitivity depends on the probe coverage of the involved genes. For example, the breakpoint of *NTRK3* fusions often occurs within a highly repetitive, intronic region, leading to high false negative rates. Indeed, Solomon et al. found a 76.9% sensitivity when evaluating *NTRK3* fusions using the MSK-IMPACT DNA-based NGS assay [160]. Conversely, RNA-NGS, including total RNA analysis, represent the optimal tools to investigate the whole fusion landscape of a tumor sample with high sensitivity and specificity. Integrated DNA/RNA-NGS assays can thus be used to achieve complete molecular profiling of a tumor and they will probably enter the diagnostic routine practice in the coming years considered the demands posed by precision medicine, of which targeting of *NTRK* fusions is an example.

Since every technique presents specific advantages and disadvantages, it is difficult to designate gold standard technique. Indeed, several algorithms have been already suggested, tailored to the different settings or tumor types. Most of them combine IHC staining as a screening tool, followed by confirmation through other techniques: following these algorithms, tumors are first evaluated by a rapid and cost-effective (but less specific) assay, allowing to focus more expensive, but highly specific tests on a smaller subset of cases [123,160,161,163,164,165,166,167]. For CNS tumors, considering the ever-increasing importance of extensive molecular profiling to achieve a correct diagnosis/classification, integration of *NTRK* fusion assessment in a dedicated NGS workflow seems desirable in the medium-term. However, as NGS availability is still limited for routine diagnostics in many centers and that IHC-screening efficacy is limited in this setting (because of a low specificity), real time-PCR assays could represent a good compromise in terms of cost-efficacy.

Finally, the optimal strategy for molecular profiling at disease progression after *NTRK* fusions-targeting should now be investigated. Present data suggest efficacy of liquid-based assessment, but given the wide range of both on-target and off-target resistance mechanisms, comprehensive assays seem to be necessary [156].

## 6. Conclusions

Management of CNS tumors represents a challenging therapeutic issue as curative surgical resection is often not feasible, and radiotherapy may have significant negative long-term consequences on neurocognitive functions (especially in children). The efficacy of chemotherapy drugs is limited, also due to the fact that blood-brain barrier considerably limits the chance of drugs to reach the tumor. Although the recognition of *NTRK* as a potential oncogene is now dated, the proper understanding of the specific mechanisms involved and their appreciation as a potential therapeutic target is far more recent. Despite the rarity of *NTRK* fusions, the potential clinical benefit for the small group of patients harboring these alterations appears to be extremely significant, thus fully awareness by physicians caring for brain tumors is now mandatory.

## Figures and Tables

**Table 1:** *NTRK* fusions in CNS tumors.

Tumor Entity	*NTRK* Fusions Frequency	Most Frequently Reported *NTRK* Fusions
Glioblastoma	1.1% (Frattini et al.) [134] 1.1% (Shah et al.) [135] 2.6% (Zheng et al.) [124] 1.2% (Kim et al.) [136] 1.7% (Ferguson et al.) [133]	*BCAN*-*NTRK1* *NFASC*-*NTRK1* *ARHGEF2*-*NTRK1* *CHTOP*-*NTRK1* *GKAP*-*NTRK2* *KCTD8*-*NTRK2* *TBC1D2*-*NTRK2* *EML4*-*NTRK3*
Non-brainstem high-grade glioma	10%–40% (Wu et al.)# [121]	*ETV6*-*NTRK3* *TPM3*-*NTRK1* *BTBD1*-*NTRK3* *VCL*-*NTRK2* *AGBL4*-*NTRK2*
DIPG°	4% (Wu et al.) [121]	
Pilocytic astrocytoma	16.6% (Ferguson et al.) [133] 3.1% (Jones et al.) [114]	*BCAN*-*NTRK1* *NACC2*-*NTRK2* *QKI*-*NTRK2*
Anaplastic astrocytoma	2.3% (Ferguson et al.) [133]	*NOS1AP*-*NTRK2*
Glioma NOS	4.1% (Ferguson et al.) [133]	*SQSTM1*-*NTRK2*
Low-grade glioma	0.7% (Zhang et al.) [112] 0.43% (Stransky et al.) [72] 4.3% (Ferguson et al.) [133]	*ETV6*-*NTRK3* *AFAP1*-*NTRK2* *VCAN*-*NTRK2*
High-grade glioneuronal tumor	Case report (Kurozumi et al.) [127]	*ARHGEF2*-*NTRK1*
Ganglioglioma	Case report (Prabhakaran et al.) [124]	*TLE4*-*NTRK2*

# Age-dependent frequency (highest rate was observed in <3yy patients). ° Diffuse Intrinsic Pontine Glioma.

**Table 2:** Main clinical trials evaluating *NTRK*-fusion inhibitors.

Molecule	Population and Enrollment	Allocation and Intervention Model	Phase	Primary Outcomes	Start Date and Current Status	Identifier
**Entrectinib** **(RXDX-101)**	Adult (minimum age: 18 Years)—84 participants	Non-Randomized—Single Group Assignment	I	Dose limiting toxicity Maximum tolerated dose Recommended Phase II dose Overall response rate	2014—Active, Not Recruiting	NCT02097810 RXDX-101-01 (STARTRK-1)
**Entrectinib** **(RXDX-101)**	Adult (minimum age: 18 Years)—300 participants (estimated)	Non-Randomized—Parallel Assignment	II	Objective response rate	2015—Recruiting	NCT02568267 RXDX-101-02 (STARTRK-2)
**Entrectinib** **(RXDX-101)**	Pediatric and Adult (maximum age: 22 Years)—65 participants	Non-Randomized—Single Group Assignment	I	Maximum tolerated dose Recommended Phase II dose Objective response rate	2016—Recruiting	NCT02650401 RXDX-101-03 (STARTRK-NG)
**Larotrectinib** **(LOXO-101)**	Pediatric and Adult (minimum age: 18 Years)—6452 participants	Non-Randomized—Parallel Assignment	II	Proportion of patients with objective response	2015—Recruiting	NCT02465060 EAY131 NCI-2015-00054
**Larotrectinib** **(LOXO-101)**	Pediatric and Adult (minimum age: 12 Years)—320 participants	Non-Randomized—Parallel Assignment	II	Best overall response rate	2015—Recruiting	NCT02576431 LOXO-TRK-15002 (NAVIGATE)
**Larotrectinib** **(LOXO-101)**	Pediatric and Adult (maximum age: 21 Years) —174 participants	Non-Randomized—Parallel Assignment	I/II	Number and severity of adverse events (Phase I) Overall response rate (Phase II)	2015—Recruiting	NCT02637687 LOXO-TRK-15003 (SCOUT)
**Larotrectinib** **(LOXO-101)**	Pediatric and Adult (from 12 Months to 21 Years)—1000 participants (estimated)	Non-Randomized—Parallel Assignment	II	Objective response rate	2017—Recruiting	NCT03155620 APEC1621SC NCI-2017-01251
**Larotrectinib** **(LOXO-101)**	Pediatric and Adult (from 12 Months to 21 Years)—49 participants	Non-Randomized—Single Group Assignment	II	Objective response rate	2017—Recruiting	NCT03213704 APEC1621A NCI-2017-01264
**Larotrectinib** **(LOXO-101)**	Pediatric and Adult (maximum age: 30 Years)—70 participants	Non-Randomized—Single Group Assignment	II	Objective response rate	2019—Recruiting	NCT03834961 ADVL1823 NCI-2019-00015
**Repotrectinib** **(TPX-0005)**	Pediatric and Adult (12 Years and older)—450 (estimated)	Non-Randomized—Single Group Assignment	I/II	Dose limiting toxicities Recommended Phase II dose Overall response rate	2019—Recruiting	NCT03093116 TPX-0005-01 (TRIDENT-1)
**Repotrectinib** **(TPX-0005)**	Pediatric (4 Years to 12 Years)—12 participants	Non-Randomized—Single Group Assignment	I	Dose limiting toxicities Pediatric recommended Phase II dose	2019—Recruiting	NCT04094610 TPX-0005-07
**Selitrectinib** **(LOXO-195)**	Pediatric and Adult (minimum age: 1 Month)	Expanded Access (Individual Patients)	NA	NA	2017—Available (Expanded Access)	NCT03206931
**Selitrectinib** **(LOXO-195)**	Pediatric and Adult (minimum age: 1 Month)—93 participants	Non-Randomized—Sequential Assignment	I/II	Maximum tolerated dose Recommended dose Overall response rate	2017—Recruiting	NCT03215511 LOXO-EXT-17005

NA: Not applicable.

**Table 3:** Available diagnostic assays for detecting *NTRK* fusions.

Assay Type	Advantages	Limitations	Turnaround Time	Main Role in Potential Diagnostic Algorithms
**IHC**	Commonly available Limited cost Minimal tissue required Allows correlation with histology Confirms protein expression panTRK antibody available	Low sensitivity or specificity in specific settings No information about the fusion partner	1–2 days	Screening
**FISH**	Minimal tissue required High sensitivity and specificity although false negative results are possible	Specific lab facilities required and expertise for interpretation No information about the fusion partner One probe-one gene evaluation, thus time-consuming and higher costs	3–5 days	Confirmatory
**RT-PCR**	Limited cost High sensitivity and specificity	Requires knowledge about the fusion partners before testing and specific primers must be prepared Good pre-analytics required to preserve RNA	5–7 days	Confirmatory
**Real time-PCR**	Limited cost High sensitivity High specificity	Good pre-analytics required to preserve RNA It does not provide information regarding the specific fusion partners and it evaluates a pre-determined set of rearrangements, thus novel or rare fusions will be missed	5–7 days	Screening/Confirmatory*
**RNA-NGS**	Evaluation of all potential fusions in a sample if Total RNA is analyzed Provides characterization of fusion partners High sensitivity High specificity	Specific lab facilities required and expertise for interpretation High costs Good pre-analytics required to preserve RNA Longer TAT	1–3 weeks	Screening/Confirmatory*
**DNA-NGS**	It can provide an overall characterization of tumor molecular profile (mutations, CNV, tumor mutation burden…) Provides characterization of fusion partners High sensitivity with some caveats High specificity	Chance of detecting non-significant chromosomal rearrangements Potential low sensitivity for specific fusions Specific lab facilities required and expertise for interpretation High costs Longer TAT	1–3 weeks	Screening/Confirmatory*
**DNA/RNA-NGS**	It provides the most complete characterization of tumor molecular profile (mutations, CNV, tumor mutation burden, fusions…) Provides characterization of fusion partners High sensitivity High specificity	Specific lab facilities required and expertise for interpretation High costs Longer TAT	1–3 weeks	Screening/Confirmatory*

* depending on each laboratory diagnostic routine workup of a sample (for instance based on tumor type) and available resources/facilities.
